# 4,5,6,7-Tetra­bromo-1,1,3-trimethyl-3-(2,3,4,5-tetra­bromo­phen­yl)indane

**DOI:** 10.1107/S1600536808000494

**Published:** 2008-01-16

**Authors:** Alex Konstantinov, Robert McCrindle, Gilles Arsenault, Alan J. Lough

**Affiliations:** a Wellington Laboratories, Research Division, Guelph, Ontario, Canada N1G 3M5; b Department of Chemistry, University of Guelph, Ontario, Canada N1G 2W1; c Department of Chemistry, University of Toronto, Ontario, Canada M5S 3H6

## Abstract

The title compound (OctaInd), C_18_H_12_Br_8_, is a commercial brominated flame retardant (BFR). In the mol­ecule, the five-membered ring has a slight envelope conformation, with a deviation of 0.317 (9) Å for the flap C atom from four essentially planar C atoms. The dihedral angle between the two benzene rings is 74.00 (16) Å.

## Related literature

For related literature, see: Andersson *et al.* (2006 ▶); Muir *et al.* (2007 ▶); Richardson (2007 ▶). See also Appendix 3 in a Danish EPA report published in 1999 on ‘Physical-chemical Properties of Brominated Flame Retardants’; http://www2.mst.dk/udgiv/Publications/1999/87-7909-416-3/html/bil03_eng.htm.
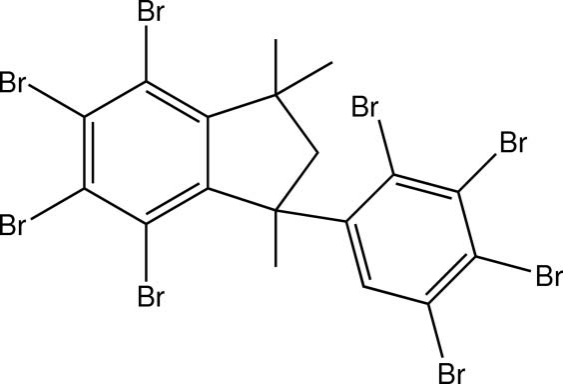


## Experimental

### 

#### Crystal data

C_18_H_12_Br_8_
*M* *_r_* = 867.56
Monoclinic, 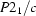

*a* = 20.2603 (6) Å
*b* = 7.3862 (2) Å
*c* = 15.2233 (8) Å
β = 110.4070 (15)°
*V* = 2135.14 (14) Å^3^
*Z* = 4
Mo *K*α radiation
μ = 15.03 mm^−1^
*T* = 150 (1) K
0.16 × 0.14 × 0.14 mm

#### Data collection

Nonius KappaCCD diffractometer
Absorption correction: multi-scan (*SORTAV*; Blessing, 1995 ▶) *T*_min_ = 0.057, *T*_max_ = 0.122
13121 measured reflections
4862 independent reflections
3509 reflections with *I* > 2σ(*I*)
*R*_int_ = 0.059

#### Refinement

*R*[*F* ^2^ > 2σ(*F* ^2^)] = 0.045
*wR*(*F* ^2^) = 0.111
*S* = 0.99
4862 reflections
238 parameters
H-atom parameters constrained
Δρ_max_ = 1.33 e Å^−3^
Δρ_min_ = −1.34 e Å^−3^

### 

Data collection: *COLLECT* (Nonius, 2002 ▶); cell refinement: *DENZO–SMN* (Otwinowski & Minor, 1997 ▶); data reduction: *DENZO–SMN*; program(s) used to solve structure: *SIR92* (Altomare *et al.*, 1994 ▶); program(s) used to refine structure: *SHELXTL* (Sheldrick, 2001 ▶); molecular graphics: *SHELXTL*; software used to prepare material for publication: *SHELXTL*.

## Supplementary Material

Crystal structure: contains datablocks global, I. DOI: 10.1107/S1600536808000494/bv2087sup1.cif

Structure factors: contains datablocks I. DOI: 10.1107/S1600536808000494/bv2087Isup2.hkl

Additional supplementary materials:  crystallographic information; 3D view; checkCIF report

## Figures and Tables

**Fig. 1.**
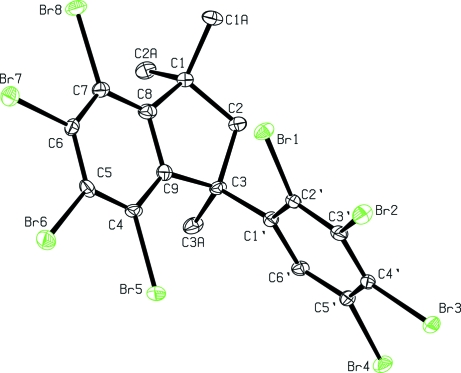
The molecular structure of the title compound. Displacement ellipsoids are at the 30% probability level. H atoms are not shown.

**Fig. 2.**
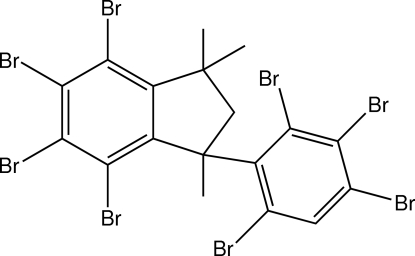
Schematic representation of 1,1,3-trimethyl-4,5,6,7-tetrabromo-3-(2,3,4,6-tetrabromophenyl)indane which is an isomer of the title compound.

## supplementary crystallographic information

### Comment

Tetrabromotrimethylphenylindane (OctaInd) is a commercial brominated flame retardant (BFR) used in styrenic and engineering thermoplastics (http://www2.mst.dk/udgiv/Publications/1999/87–7909-416–3/html/bil03_eng.htm). The major component in the commercial mixture is believed to be 1,1,3-trimethyl-4,5,6,7-tetrabromo-3-(2,3,4,5-tetrabromophenyl)indane. BFRs have been used in a variety of products to protect human life and property against fires. However, there is a growing concern that these BFR compounds are becoming significant environmental contaminants because of their widespread presence in the environment and in human and wildlife samples (Richardson, 2007). Very little is known about OctaInd and, to the best of our knowledge, it has not been reported in the environmental literature. However, OctaInd was one of the top ten persistent brominated or chlorinated compounds identified by QSPR screening that deserves greater attention (Muir *et al.*, 2007). In a recent modeling study (Andersson *et al.*, 2006) OctaInd was described as being 1,1,3-trimethyl-4,5,6,7-tetrabromo-3-(2,3,4,6-tetrabromophenyl)indane (note the different substitution pattern on the C1'-C6' ring in Fig 2) but our X-ray structure determination estabilshed that OctaInd has the structure shown in Fig. 1. This information is important to researchers wishing to model the behaviour of OctaInd.

### Experimental

1,1,3-trimethyl-4,5,6,7-tetrabromo-3-(2,3,4,5-tetrabromophenyl)indane was obtained by bromination of 1,1,3-trimethyl-3-phenylindane using proprietary methods. The compound was isolated using chromatographic techniques. Colorless crystals were obtained from a solution of the title compound in toluene.

### Refinement

All hydrogen atoms were placed in calculated positions with C—H distances of 0.98 and 0.99 Å and they were included in the refinement in a riding-model approximation with *U*_iso_ = 1.2*U*_eq_(C) or 1.5*U*_eq_(C) for methyl C atoms.

### Crystal data

**Table:**

C_18_H_12_Br_8_	*F*_000_ = 1600
*M**_r_* = 867.56	*D*_x_ = 2.699 Mg m^−^^3^
Monoclinic, *P*2_1_/*c*	Mo *K*α radiation λ = 0.71073 Å
Hall symbol: -P 2ybc	Cell parameters from 13121 reflections
*a* = 20.2603 (6) Å	θ = 2.7–27.5º
*b* = 7.3862 (2) Å	µ = 15.03 mm^−^^1^
*c* = 15.2233 (8) Å	*T* = 150 (1) K
β = 110.4070 (15)º	Block, colourless
*V* = 2135.14 (14) Å^3^	0.16 × 0.14 × 0.14 mm
*Z* = 4	

### Data collection

**Table:**

Nonius KappaCCD diffractometer	4862 independent reflections
Radiation source: fine-focus sealed tube	3509 reflections with *I* > 2σ(*I*)
Monochromator: graphite	*R*_int_ = 0.059
Detector resolution: 9 pixels mm^-1^	θ_max_ = 27.5º
*T* = 150(1) K	θ_min_ = 2.7º
φ scans and ω scans with κ offsets	*h* = −25→26
Absorption correction: multi-scan(SORTAV; Blessing, 1995)	*k* = −9→8
*T*_min_ = 0.057, *T*_max_ = 0.122	*l* = −19→19
13121 measured reflections	

### Refinement

**Table:**

Refinement on *F*^2^	Secondary atom site location: difference Fourier map
Least-squares matrix: full	Hydrogen site location: inferred from neighbouring sites
*R*[*F*^2^ > 2σ(*F*^2^)] = 0.045	H-atom parameters constrained
*wR*(*F*^2^) = 0.111	*w* = 1/[σ^2^(*F*_o_^2^) + (0.0587*P*)^2^] where *P* = (*F*_o_^2^ + 2*F*_c_^2^)/3
*S* = 0.99	(Δ/σ)_max_ = 0.001
4862 reflections	Δρ_max_ = 1.33 e Å^−^^3^
238 parameters	Δρ_min_ = −1.34 e Å^−^^3^
Primary atom site location: structure-invariant direct methods	Extinction correction: none

### Special details

**Table:**

Geometry. All e.s.d.'s (except the e.s.d. in the dihedral angle between two l.s. planes) are estimated using the full covariance matrix. The cell e.s.d.'s are taken into account individually in the estimation of e.s.d.'s in distances, angles and torsion angles; correlations between e.s.d.'s in cell parameters are only used when they are defined by crystal symmetry. An approximate (isotropic) treatment of cell e.s.d.'s is used for estimating e.s.d.'s involving l.s. planes.
Refinement. Refinement of *F*^2^ against ALL reflections. The weighted *R*-factor *wR* and goodness of fit *S* are based on *F*^2^, conventional *R*-factors *R* are based on *F*, with *F* set to zero for negative *F*^2^. The threshold expression of *F*^2^ > σ(*F*^2^) is used only for calculating *R*-factors(gt) *etc*. and is not relevant to the choice of reflections for refinement. *R*-factors based on *F*^2^ are statistically about twice as large as those based on *F*, and *R*- factors based on ALL data will be even larger.

### Fractional atomic coordinates and isotropic or equivalent isotropic displacement parameters (Å^2^)

**Table:**

	*x*	*y*	*z*	*U*_iso_*/*U*_eq_	
Br1	0.27614 (3)	0.65858 (8)	0.41094 (4)	0.02811 (17)	
Br2	0.42084 (3)	0.88707 (8)	0.48485 (5)	0.03059 (17)	
Br3	0.56522 (3)	0.70567 (8)	0.63052 (4)	0.02834 (17)	
Br4	0.55454 (3)	0.31103 (9)	0.72840 (4)	0.03124 (17)	
Br5	0.28644 (3)	0.57820 (9)	0.70187 (4)	0.03205 (17)	
Br6	0.14076 (4)	0.81344 (9)	0.65538 (5)	0.03543 (19)	
Br7	0.01011 (3)	0.75190 (9)	0.45738 (5)	0.03139 (17)	
Br8	0.01921 (3)	0.43423 (9)	0.31278 (4)	0.03253 (18)	
C1	0.1758 (3)	0.2215 (7)	0.3861 (4)	0.0219 (13)	
C1A	0.1588 (4)	0.2594 (8)	0.2807 (4)	0.0288 (15)	
H1AA	0.1810	0.3735	0.2731	0.043*	
H1AB	0.1077	0.2685	0.2493	0.043*	
H1AC	0.1770	0.1604	0.2529	0.043*	
C1'	0.3483 (3)	0.3945 (7)	0.5524 (4)	0.0206 (13)	
C2	0.2562 (3)	0.2027 (8)	0.4324 (4)	0.0226 (13)	
H2A	0.2800	0.2652	0.3940	0.027*	
H2B	0.2698	0.0733	0.4372	0.027*	
C2A	0.1361 (3)	0.0495 (7)	0.3963 (5)	0.0294 (15)	
H2AA	0.0853	0.0730	0.3723	0.044*	
H2AB	0.1508	0.0155	0.4626	0.044*	
H2AC	0.1469	−0.0495	0.3606	0.044*	
C2'	0.3537 (3)	0.5593 (7)	0.5085 (4)	0.0205 (12)	
C3	0.2792 (3)	0.2890 (7)	0.5320 (4)	0.0212 (13)	
C3A	0.2846 (3)	0.1397 (8)	0.6056 (4)	0.0294 (15)	
H3AA	0.3016	0.1929	0.6685	0.044*	
H3AB	0.3175	0.0459	0.6012	0.044*	
H3AC	0.2381	0.0858	0.5935	0.044*	
C3'	0.4168 (3)	0.6534 (8)	0.5333 (4)	0.0240 (13)	
C4	0.2098 (3)	0.5358 (8)	0.5891 (4)	0.0249 (14)	
C4'	0.4786 (3)	0.5817 (7)	0.5981 (4)	0.0201 (13)	
C5	0.1486 (3)	0.6364 (8)	0.5695 (4)	0.0253 (14)	
C5'	0.4740 (3)	0.4165 (8)	0.6388 (4)	0.0232 (13)	
C6	0.0929 (3)	0.6090 (8)	0.4852 (4)	0.0229 (13)	
C6'	0.4110 (3)	0.3272 (7)	0.6172 (4)	0.0208 (13)	
H6'A	0.4099	0.2153	0.6474	0.025*	
C7	0.0975 (3)	0.4758 (8)	0.4235 (4)	0.0232 (13)	
C8	0.1586 (3)	0.3757 (8)	0.4420 (4)	0.0244 (13)	
C9	0.2156 (3)	0.4084 (7)	0.5251 (4)	0.0222 (13)	

### Atomic displacement parameters (Å^2^)

**Table:**

	*U*^11^	*U*^22^	*U*^33^	*U*^12^	*U*^13^	*U*^23^
Br1	0.0233 (3)	0.0283 (3)	0.0269 (3)	−0.0021 (3)	0.0014 (3)	0.0100 (2)
Br2	0.0284 (4)	0.0269 (3)	0.0339 (4)	−0.0043 (3)	0.0076 (3)	0.0071 (3)
Br3	0.0214 (3)	0.0361 (4)	0.0255 (4)	−0.0062 (3)	0.0058 (3)	0.0005 (3)
Br4	0.0226 (3)	0.0402 (4)	0.0257 (4)	0.0027 (3)	0.0019 (3)	0.0090 (3)
Br5	0.0270 (4)	0.0470 (4)	0.0193 (3)	−0.0080 (3)	0.0046 (3)	−0.0090 (3)
Br6	0.0361 (4)	0.0397 (4)	0.0333 (4)	−0.0052 (3)	0.0155 (3)	−0.0145 (3)
Br7	0.0281 (4)	0.0332 (4)	0.0333 (4)	0.0045 (3)	0.0112 (3)	0.0007 (3)
Br8	0.0250 (4)	0.0405 (4)	0.0239 (4)	0.0027 (3)	−0.0018 (3)	−0.0040 (3)
C1	0.021 (3)	0.026 (3)	0.018 (3)	0.000 (2)	0.005 (3)	0.000 (2)
C1A	0.035 (4)	0.033 (3)	0.016 (3)	−0.003 (3)	0.005 (3)	0.000 (2)
C1'	0.019 (3)	0.022 (3)	0.020 (3)	−0.003 (2)	0.006 (3)	−0.001 (2)
C2	0.022 (3)	0.023 (3)	0.020 (3)	0.001 (2)	0.003 (3)	−0.002 (2)
C2A	0.029 (4)	0.027 (3)	0.029 (4)	−0.005 (3)	0.005 (3)	−0.005 (3)
C2'	0.022 (3)	0.024 (3)	0.015 (3)	−0.002 (2)	0.007 (2)	−0.001 (2)
C3	0.019 (3)	0.024 (3)	0.017 (3)	−0.008 (2)	0.002 (2)	0.002 (2)
C3A	0.025 (3)	0.031 (3)	0.026 (4)	−0.004 (3)	0.002 (3)	0.007 (3)
C3'	0.026 (3)	0.029 (3)	0.017 (3)	−0.002 (3)	0.007 (3)	0.002 (2)
C4	0.023 (3)	0.033 (3)	0.016 (3)	−0.015 (3)	0.004 (3)	−0.005 (2)
C4'	0.019 (3)	0.024 (3)	0.018 (3)	−0.003 (2)	0.007 (2)	−0.006 (2)
C5	0.031 (4)	0.027 (3)	0.023 (3)	−0.003 (3)	0.016 (3)	−0.005 (3)
C5'	0.018 (3)	0.030 (3)	0.020 (3)	0.006 (2)	0.005 (3)	0.001 (2)
C6	0.017 (3)	0.026 (3)	0.026 (3)	−0.002 (2)	0.008 (3)	−0.001 (2)
C6'	0.023 (3)	0.020 (3)	0.018 (3)	−0.001 (2)	0.004 (2)	0.004 (2)
C7	0.023 (3)	0.025 (3)	0.020 (3)	−0.008 (3)	0.005 (3)	0.003 (2)
C8	0.027 (3)	0.026 (3)	0.019 (3)	−0.004 (3)	0.005 (3)	0.003 (2)
C9	0.023 (3)	0.024 (3)	0.021 (3)	−0.008 (3)	0.009 (3)	0.002 (2)

### Geometric parameters (Å, °)

**Table:**

Br1—C2'	1.894 (6)	C2—H2B	0.9900
Br2—C3'	1.890 (6)	C2A—H2AA	0.9800
Br3—C4'	1.886 (6)	C2A—H2AB	0.9800
Br4—C5'	1.892 (6)	C2A—H2AC	0.9800
Br5—C4	1.897 (6)	C2'—C3'	1.387 (8)
Br6—C5	1.895 (6)	C3—C9	1.533 (8)
Br7—C6	1.900 (6)	C3—C3A	1.548 (8)
Br8—C7	1.895 (6)	C3A—H3AA	0.9800
C1—C8	1.533 (8)	C3A—H3AB	0.9800
C1—C2	1.540 (8)	C3A—H3AC	0.9800
C1—C2A	1.541 (8)	C3'—C4'	1.401 (8)
C1—C1A	1.544 (8)	C4—C5	1.386 (9)
C1A—H1AA	0.9800	C4—C9	1.390 (8)
C1A—H1AB	0.9800	C4'—C5'	1.386 (8)
C1A—H1AC	0.9800	C5—C6	1.396 (8)
C1'—C6'	1.400 (8)	C5'—C6'	1.371 (8)
C1'—C2'	1.411 (8)	C6—C7	1.386 (8)
C1'—C3	1.537 (8)	C6'—H6'A	0.9500
C2—C3	1.560 (8)	C7—C8	1.384 (8)
C2—H2A	0.9900	C8—C9	1.405 (8)
			
C8—C1—C2	102.8 (5)	C3—C3A—H3AA	109.5
C8—C1—C2A	109.2 (5)	C3—C3A—H3AB	109.5
C2—C1—C2A	112.7 (5)	H3AA—C3A—H3AB	109.5
C8—C1—C1A	115.5 (5)	C3—C3A—H3AC	109.5
C2—C1—C1A	107.9 (5)	H3AA—C3A—H3AC	109.5
C2A—C1—C1A	108.7 (5)	H3AB—C3A—H3AC	109.5
C1—C1A—H1AA	109.5	C2'—C3'—C4'	121.0 (5)
C1—C1A—H1AB	109.5	C2'—C3'—Br2	120.8 (4)
H1AA—C1A—H1AB	109.5	C4'—C3'—Br2	118.1 (4)
C1—C1A—H1AC	109.5	C5—C4—C9	120.0 (5)
H1AA—C1A—H1AC	109.5	C5—C4—Br5	119.7 (4)
H1AB—C1A—H1AC	109.5	C9—C4—Br5	120.2 (5)
C6'—C1'—C2'	116.1 (5)	C5'—C4'—C3'	117.7 (5)
C6'—C1'—C3	120.1 (5)	C5'—C4'—Br3	120.8 (4)
C2'—C1'—C3	123.9 (5)	C3'—C4'—Br3	121.5 (4)
C1—C2—C3	108.5 (5)	C4—C5—C6	119.8 (5)
C1—C2—H2A	110.0	C4—C5—Br6	120.3 (5)
C3—C2—H2A	110.0	C6—C5—Br6	119.9 (5)
C1—C2—H2B	110.0	C6'—C5'—C4'	121.3 (5)
C3—C2—H2B	110.0	C6'—C5'—Br4	118.1 (4)
H2A—C2—H2B	108.4	C4'—C5'—Br4	120.6 (4)
C1—C2A—H2AA	109.5	C7—C6—C5	120.1 (5)
C1—C2A—H2AB	109.5	C7—C6—Br7	120.4 (4)
H2AA—C2A—H2AB	109.5	C5—C6—Br7	119.5 (4)
C1—C2A—H2AC	109.5	C5'—C6'—C1'	122.5 (5)
H2AA—C2A—H2AC	109.5	C5'—C6'—H6'A	118.7
H2AB—C2A—H2AC	109.5	C1'—C6'—H6'A	118.7
C3'—C2'—C1'	121.3 (5)	C8—C7—C6	120.6 (5)
C3'—C2'—Br1	117.0 (4)	C8—C7—Br8	120.4 (4)
C1'—C2'—Br1	121.7 (4)	C6—C7—Br8	119.0 (5)
C9—C3—C1'	114.0 (4)	C7—C8—C9	119.2 (6)
C9—C3—C3A	107.8 (5)	C7—C8—C1	130.1 (5)
C1'—C3—C3A	112.7 (5)	C9—C8—C1	110.7 (5)
C9—C3—C2	102.1 (4)	C4—C9—C8	120.2 (6)
C1'—C3—C2	110.2 (5)	C4—C9—C3	128.0 (5)
C3A—C3—C2	109.5 (5)	C8—C9—C3	111.8 (5)

## References

[bb1] Altomare, A., Cascarano, G., Giacovazzo, C., Guagliardi, A., Burla, M. C., Polidori, G. & Camalli, M. (1994). *J. Appl. Cryst.* **27**, 435.

[bb2] Andersson, P. L., Öberg, K. & Örn, U. (2006). *Environ. Toxicol. Chem.* **25**, 1275–1282. 10.1897/05-342r.1 16704058

[bb3] Blessing, R. H. (1995). *Acta Cryst.* A**51**, 33–38. 10.1107/s0108767394005726 7702794

[bb4] Muir, D., Howard, P. H. & Metlan, W. (2007). *Organohalogen Compd.* **69**, 1053–1056.

[bb5] Nonius (2002). *COLLECT* Nonius BV, Delft, The Netherlands.

[bb6] Otwinowski, Z. & Minor, W. (1997). *Methods in Enzymology*, Vol. 276, *Macromolecular Crystallography*, Part A, edited by C. W. Carter Jr & R. M. Sweet, pp. 307–326. New York: Academic Press.

[bb7] Richardson, S. D. (2007). *Anal. Chem.* **79**, 4295–4324. 10.1021/ac070719q 17508722

[bb8] Sheldrick, G. M. (2001). *SHELXTL/PC* Version 6.1. Windows XP Version. Bruker AXS Inc., Madison, USA.

